# Elucidation of the Clustered Nano-Architecture of Radiation-Induced DNA Damage Sites and Surrounding Chromatin in Cancer Cells: A Single Molecule Localization Microscopy Approach

**DOI:** 10.3390/ijms22073636

**Published:** 2021-03-31

**Authors:** Michael Hausmann, Martin Falk, Charlotte Neitzel, Andreas Hofmann, Abin Biswas, Theresa Gier, Iva Falkova, Dieter W. Heermann, Georg Hildenbrand

**Affiliations:** 1 Kirchhoff Institute for Physics, Heidelberg University, 69120 Heidelberg, Germany; charlotte.neitzel@kip.uni-heidelberg.de (C.N.); abinbiswas@gmail.com (A.B.); theresa@fam-gier.de (T.G.); hilden@kip.uni-heidelberg.de (G.H.); 2 Institute of Biophysics, Czech Academy of Sciences, 612 65 Brno, Czech Republic; ivafalk@seznam.cz; 3 Institute for Theoretical Physics, Heidelberg University, 69120 Heidelberg, Germany; AH@andreashofmann.org (A.H.); heermann@tphys.uni-heidelberg.de (D.W.H.)

**Keywords:** topology of DNA double strand breaks, nano-architecture, ionizing radiation-induced foci (IRIF), chromatin rearrangements after irradiation, single molecule localization microscopy (SMLM)

## Abstract

In cancer therapy, the application of (fractionated) harsh radiation treatment is state of the art for many types of tumors. However, ionizing radiation is a “double-edged sword”—it can kill the tumor but can also promote the selection of radioresistant tumor cell clones or even initiate carcinogenesis in the normal irradiated tissue. Individualized radiotherapy would reduce these risks and boost the treatment, but its development requires a deep understanding of DNA damage and repair processes and the corresponding control mechanisms. DNA double strand breaks (DSBs) and their repair play a critical role in the cellular response to radiation. In previous years, it has become apparent that, beyond genetic and epigenetic determinants, the structural aspects of damaged chromatin (i.e., not only of DSBs themselves but also of the whole damage-surrounding chromatin domains) form another layer of complex DSB regulation. In the present article, we summarize the application of super-resolution single molecule localization microscopy (SMLM) for investigations of these structural aspects with emphasis on the relationship between the nano-architecture of radiation-induced repair foci (IRIFs), represented here by γH2AX foci, and their chromatin environment. Using irradiated HeLa cell cultures as an example, we show repair-dependent rearrangements of damaged chromatin and analyze the architecture of γH2AX repair clusters according to topological similarities. Although HeLa cells are known to have highly aberrant genomes, the topological similarity of γH2AX was high, indicating a functional, presumptively genome type-independent relevance of structural aspects in DSB repair. Remarkably, nano-scaled chromatin rearrangements during repair depended both on the chromatin domain type and the treatment. Based on these results, we demonstrate how the nano-architecture and topology of IRIFs and chromatin can be determined, point to the methodological relevance of SMLM, and discuss the consequences of the observed phenomena for the DSB repair network regulation or, for instance, radiation treatment outcomes.

## 1. Introduction

The DNA organized into the chromatin in the eukaryotic cell nucleus is permanently attacked and damaged by environmental factors such as chemicals and drugs [1,2,3,4] or, for instance, UV or ionizing radiation (e.g., X-rays, particles of atomic decays, etc.) [5,6]. Such damage may dramatically impact intracellular processes such as energy metabolism, DNA replication or protein synthesis (see, for example, [4,7,8,9,10]). Hence, the cells would never properly function if they would not have developed efficient strategies and mechanisms to repair all types of DNA damage. A properly regulated and quickly functioning DNA repair network [11,12,13,14,15,16,17,18,19,20,21] is therefore a prerequisite for normal cell life and survival.

Individual biochemical processes and their sequences (repair pathways) [14,16] operating in cells to maintain genome integrity have been described in detail (for review, e.g., [21]). In the context of DSB repair, several repair pathways can be distinguished based on the requirement for the DNA-end resection and presence of homologous repair templates: (a) non-homologous end-joining (NHEJ) [17,18,22,23,24], the fast and seemingly most often used pathway in mammals; (b) homologous recombination (HR) [19,23,25,26,27], the error-free but slower pathway; and (c) alternative or back-up end-joining mechanisms (a-Ej) [28,29,30,31,32], whose classification is not yet entirely obvious as they combine aspects of both NHEJ and HR to varying degrees. Single-chain hybridization (single-strand annealing, SSA) [31] and microhomology-mediated end-joining (MMEJ) [33,34] can be mentioned as commonly appearing as A-Ej in the literature.

However, the relationship between NHEJ, HR, and A-Ej pathways still remains mysterious, especially the mechanism through which a cell makes the decision for a specific pathway at each given DSB site, which is still subjected to further investigation [13,14,16,20,35,36,37,38]. This general question may also be interpreted as how certain repair proteins—in contrast to others—gain favorable access to DSB sites and thus may initiate a certain repair pathway. In other words, is there some defined spatial organization of chromatin that could be correlated with molecular mechanisms of DNA damage formation and/or repair [21,39]? Answering these questions is important to understand the outcome of damage and repair processes with respect to cell survival and the risk of mutagenesis. In turn, a better understanding of DSB repair regulation will allow the targeted modification of repair process activity under given irradiation or biological conditions; thus offering an important and highly versatile therapeutic approach, the applicable of personalized cancer radiotherapy or chemotherapy [40,41,42].

In the present manuscript, we hypothesize that the original architecture (packaging, composition, molecular accessibility, etc.) of a damaged chromatin domain and its near and far environment fundamentally affects the kinetics of DSB manifestation and repair. This idea is supported by recent findings that initially condensed heterochromatin domains damaged by radiation must undergo extensive decondensation prior to DSB repair commencing [43,44]. On the other hand, damaged loci in euchromatin seem to be converted to a more compact architecture [44]. Moreover, repair can be successfully accomplished only if the altered structure and epigenetic status of chromatin is restored to the original state [45,46,47,48]. The need for these reorganization steps may explain or at least contribute to a slower course of DSB repair in heterochromatin [49,50,51,52]. These findings point to the crucial role of the as yet unexplored aspects of DSB repair, namely its organization in space and time and regulation through the structure of chromatin.

In order to investigate the hypothesized mechanisms and to apply the basic findings to cancer research and diagnosis, a comprehensive toolbox is necessary to researchers that will allow to routinely provide an insight into spatial chromatin organization and determine cell type-specific or individual differences and potential treatment outcomes. Light and confocal microscopy—as unique methods in radiobiological research—have brought extensive knowledge of the organization of repair processes in space and time [11,51,52,53]. However, to obtain mechanistic insights into the true nature of spatial organization behind DNA damaging, repair, and misrepair, we must delve deeper into the level of interactions between individual molecules [54,55,56,57,58,59,60,61]. While confocal microscopy makes it possible to monitor the formation and dynamic evolution of IRIF and, to a certain extent, their (micro)architecture and (micro)architecture of the surrounding chromatin, the optical resolution offered is insufficient for a detailed description of these objects, their internal composition, and related processes at the molecular level [21,51,52,58]. Until recently, the only available technique for studying DSB repair at the nanoscale was electron microscopy, which offers unprecedented resolution [49,62,63] but is doing so at the expense of relatively destructive specimen fixation and requires specimens to be mechanically cut into ultrathin sections [64].

Therefore, the invention of pioneering methods of super-resolution optical microscopy [65,66], which in various ways managed to circumvent the Abbe diffraction-based resolution limit and brought the resolution of fluorescence light microscopy to the limit of about one molecule (order of 10 nm), can be considered as a breakthrough in cell biology and radiobiology (see, e.g., [38,54,55,57,58,67,68,69,70]). Although these optical techniques do not reach the resolution values of electron microscopy in practical, routine biological applications, they do allow quantitative measurements of the individual positioning of molecules and, at the same time, retain most of the advantages of standard optical microscopy. Hence, to address key radiobiological issues, we introduced single molecule localization microscopy (SMLM) and proposed compatible evaluation procedures applicable to radiation and cancer research and diagnosis.

SMLM is one of the recently developed methods of super-resolution microscopy derived from spectral precision distance microscopy which was invented about 25 years ago [71,72,73]. In addition to the molecular optical resolution, the huge advantage of SMLM can be seen in its user friendliness and application versatility—the method utilizes the same technique of sample preparation and labeling as the standard optical widefield/confocal microscopy [54,55,58,67,74]. The only indispensable requirement for specimen preparation is the use of fluorochromes that must “blink” after the initial high-energy laser illumination; i.e., the fluorochrome molecules must exert reversible photobleaching during data acquisition [75,76]. This possibility of “routine” immunofluorescence staining provides a unique opportunity to study the same samples by using standard widefield/confocal microscopy and SMLM, and thereby obtain correlated results for given specimens on the micro-, meso-, and nano-scale level [54,58,67].

In this article, we want to overview and present a summary on how SMLM and mathematical evaluation procedures of SMLM datasets can be applied for studies of the spatial (nano)architecture of chromatin damage sites, their chromatin environment, and rearrangements provoked by specific irradiation conditions and repair processes. The aim of the investigations described here is to characterize spatial, geometric parameters and the topology (see the definition below) of single repair foci in the cell nucleus and to explore how all these parameters depend on the physical characteristics of the incident radiation, focus location within a particular chromatin environment, focus type (given by the repair protein involved) and repair mechanism activated. Note: In the following, the term “topology” is used in the sense as it is defined in mathematics; i.e., it is not directly related to TADs (topologically associated domains) as in genetics. In mathematics, topology (from Greek: the study of location) is concerned with the properties of a geometric object. These properties persist mostly as scale invariant; i.e., they are preserved under certain deformations, such as stretching, twisting, certain types of crumpling, and bending. However, tearing into sub-objects or gluing to higher structures are excluded.

## 2. Results

### 2.1. From Widefield Light Microscopy to SMLM Data Acquisition

**Figure 1:**
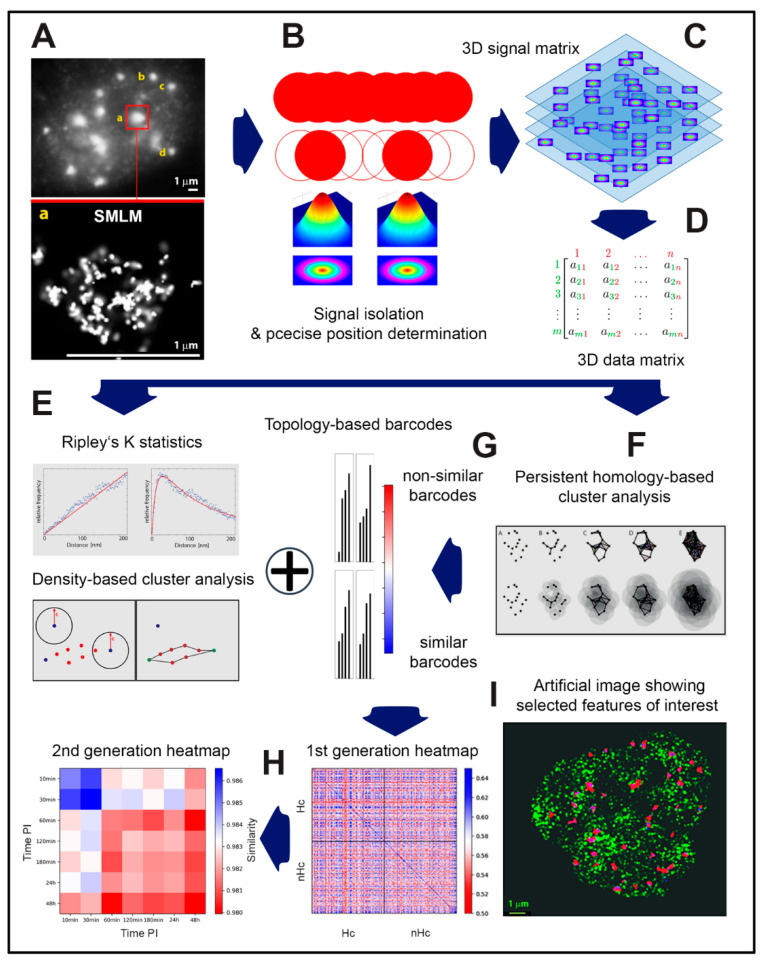
SMLM data acquisition and analysis procedure. After acquiring of a wide field image (**A** = part of Figure 2 for illustration; for further details, subfigures and scale bar see figure legend there), fluorochromes specifically attached to the targets are “reversibly” bleached by a powerful laser pulse. Subsequently, individual fluorochromes (labeling individual molecules of interest) randomly return to the fluorescent state, quench again, and then repeatedly oscillate between these states; i.e., they blink. Acquisition of a time series of several hundred to thousand image frames (**B**) allows registration of individual signals in a time sequence and thus their separation in space. This separation enables precise localization of individual signals with a precision far below the Abbe limit (currently up to 10 nm) (**C**). The localization of each isolated image point (i.e., localization of its intensity barycenter) (**C**) provides a 2D or 3D matrix of point signals that can be easily converted to a 3D coordinate matrix (extended for some other signal parameters, such as localization error) (**D**). The obtained 3D coordinate matrix is processed by various mathematical approaches, including, for instance, Ripley’s K statistics (also see for comparison Figure 3), density-based cluster analysis (**E**; Note: This figure is a modified part of a figure originally published under CC BY licence in [67]) and persistent homology-based cluster analysis (**F**; Note: This figure is a modified part of a figure originally published under CC BY licence in [79]). By these mathematical approaches, nanostructures (repair protein complexes, receptors, specific chromatin domains, etc.) are identified and described in a topologically invariant way. Topology in the sense of mathematics means that the results are not influenced by the orientation or scale deformation of nanostructures, thus allowing mutual (topological) comparison of individual nanostructures within a particular cell (nucleus) as well as among cells (cell nuclei) (**F**). In addition, using persistence homology analysis, 3D objects can easily be described by one dimensional barcodes (**G**) that can easily be compared, e.g., in the form of heatmaps. The similarity of individual barcodes can be mathematically evaluated and expressed in terms of similarity values (for instance, Jaccard index between 0 and 1) and graphically coded by a color gradient. By comparing each nanostructure versus each nanostructure, so-called 1st generation heatmaps ((**H**), right) can be constructed, giving a visual summary of topological relationships between all nanostructures. For instance, mutual similarity of DSB repair complexes (IRIFs) can be studied in dependence of their localization in heterochromatin or euchromatin ((**H**), right). By averaging the heatmaps for all nanostructures within a particular sample (e.g., a given IR dose, PI time, etc.), 2nd generation heatmaps ((**H**), left) can be computed that visualize nanostructure topological changes in dependence of various factors. Based on mathematically processed data matrices, artificial images can be generated that emphasize various features of interest, such as IRIF formation, colocalization, etc., without a need for complicated image data analysis; heterochromatin (green) and γH2AX foci (red), blue signals correspond to signal overlap of the two channels. (**I**). Note: For Figure 1E–H, examples and applications are shown in the results.

SMLM (Figure 1) is based on a very simple experience known for decades in astronomy. Depending on the signal to light background ratio, the localization of a point correlating to a maximum or minimum intensity value (i.e., positive or negative peak of the intensity distribution of the detected signal) can be determined far more precisely than the distance between two separated points (i.e., the distance of two peaks of signal intensity distributions) [71,77,78]. So, the trick for microscopy is to separate the signals of individual fluorochromes in time and thus in space [67,74] (Figure 1A,B). In brief, the procedure involves the following steps: First, a standard image of the object of interest—here, for instance, a cell nucleus with immuno-fluorescently labeled histones γH2AX—is recorded using standard widefield microscopy (Figure 1A). This image provides us with basic spatial properties and the quality of the selected objects on the micro-scale (the size and shape of the nucleus, approximate number and distribution of repair foci, overall quality of staining, etc.).

**Figure 2:**
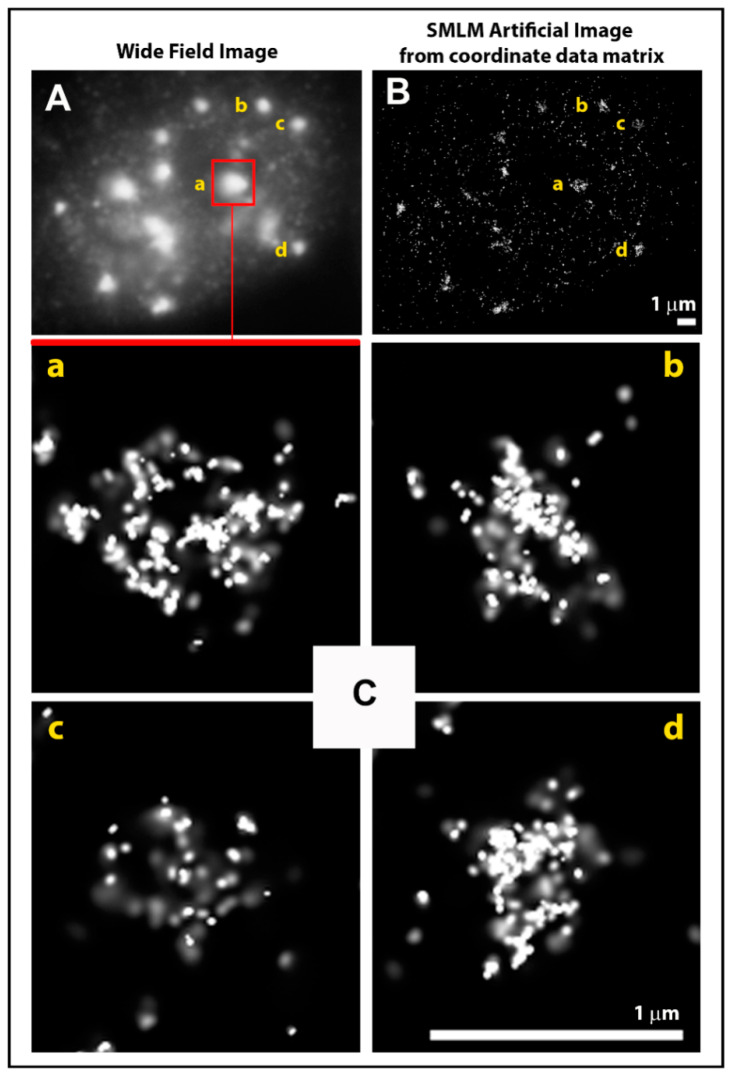
Visualization of γH2AX in the same cell nucleus using standard immuno-fluorescence microscopy (**A**) and single-molecule localization microscopy (SMLM) (**B**). Image B was created artificially based on a coordinate matrix obtained from 4000 SMLM image frames over time. (**Ca**–**Cd**) Detailed depiction of the pointillist nanostructure of selected γH2AX molecular clusters from panels (**A**,**B**) annotated by a–d. Note: Parts of this figure are shown in Figure 1A for further illustration.

All fluorochrome molecules light up simultaneously and their signals overlap due to diffraction of the microscope lens (Figure 1B, top). Because of this signal interference, only normal resolution can be achieved as defined by the Rayleigh–Abbe criterion [80] (Figure 1A and Figure 2a). In the next step, the microscope is switched to the SMLM mode and the sample is illuminated with a strong laser pulse (in the order of kW/cm^2^). Such a high laser light power makes organic fluorescent dyes and fluorescent proteins undergo so-called reversible photobleaching [75,76], which means they are stochastically transferred into a temporarily unlit state, from which they accidentally switch back to a lit, fluorescent state—they flash or “blink”. At any given moment, only a small number of fluorochromes switch between the off and the on state of blinking, giving us the opportunity to isolate their signals in time and space (Figure 1B). Hence, there is no more interference between signals, so the intensity profile of each of them can be fitted by a Gaussian function and the position of the signal barycenter can be determined with very high precision (Figure 1C). By taking a time series of several thousand image frames of the observed object with a high frequency of >10 Hz (<100 ms per frame), a matrix is obtained of exact coordinates of all signals and their other parameters, including measurement errors (Figure 1D). These matrices can be directly subjected to a variety of mathematical calculations (see below) without the necessity of often complex and time demanding image analysis (Figure 1E–H). On the other hand, one can construct an artificial image of a pointillist character from both raw and mathematically processed data to visually demonstrate the revealed phenomena (Figure 1I and Figure 2) [54,55,58,67]. It should be noted that even though the pointillist image (Figure 1I and Figure 2(Cb,Cc)) feels less natural to an inexperienced eye and seems to yield less information compared to the “standard” image, the opposite is in fact true. The pointillist image is much closer to reality because it does not contain any noise caused by signal interference, the noise to which our brains have become accustomed.

### 2.2. SMLM Data Simulations and Interpretation

Super-resolution light microscopy is often understood as light microscopy with a resolution improved down to the dimensions of single molecules (in the order of 10 nm). However, such a view is very reductive and overlooks the main benefits of super-resolution techniques, as emphasized below. Moreover, scientists who are used to interpreting results only on visual impression may be confused by pointillist (SMLM) images, which leave in doubt whether the incredibly detailed observed structures are real or artificial. For instance, the colocalization of biomolecules (proteins, enzymes, antigens etc.) is an established prerequisite for their molecular interaction. In diffraction-limited fluorescence microscopy, colocalization can be intuitively detected as the merging of two fluorescent colors into a mixed one; e.g., colocalized red and green labeling spots give rise to a yellow spot. Physics of diffraction, however, tells us that even with high numerical aperture objective lenses, such the merging or mixing of colors, there is the visual consequence of diffractive mixing. It always occurs if the distance between two labeled molecules is below the resolution limit (in fluorescence light microscopy, about 200 nm)—the full width at half maximum of the point spread function. In the case of 10-nm super-resolution conditions, a 200-nm-sized region will be resolved in a couple of independent single molecules so that the information of molecular interaction obtained by apparent colocalization seems to get lost. Thus, at first glance, additional scientific results that may justify these advanced and cost-intensive super-resolution techniques may not be obvious, especially in the case of pointillist SMLM images visually resolving any coherent structures in independent points.

In the following, we present how, instead of processed images, mathematical procedures and algorithms applied on the raw coordinate data of the detected specific labeling points can lead to a better quantitative understanding of chromatin rearrangements and repair protein recruitment and trafficking, thereby elucidating spatial conformation-driven processes, potentially also involving repair pathway decision-making procedures. The basic information for all further calculations and biological mechanistic interpretations is a table of coordinates (±measurement errors) of molecular blinking events extracted from the acquired time stack of image frames. To examine the spatial architecture and configurations of the studied labeled molecules (markers of DSBs, heterochromatin, etc.), pairwise point distances and their frequencies are analyzed. A well-established procedure for this purpose is the application of Ripley’s pattern analysis for structuring criteria [10,67,81,82]. The number of distances from each point to each other point is plotted in a frequency histogram. The shape of such histograms reflects the structural organization within the point pattern acquired, as explained in Figure 2.

**Figure 3:**
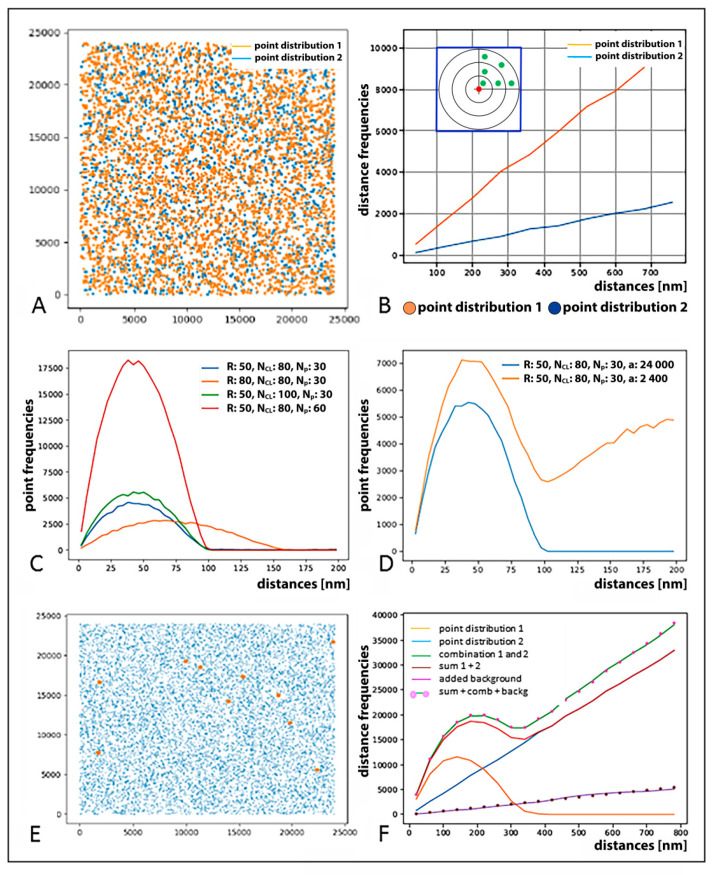
(**A**) Computer simulation of artificial point patterns and related Ripley evaluation curves used to demonstrate how structures and their descriptions can be extracted from distance frequency histogram curves. (**A**) Example of a point pattern of two homogenous (random) point distributions (blue 2 × 10^3^ points, orange 4 × 10^3^ points) within an area of 24,000 × 24,000 nm^2^. (**B**) Distance frequency distribution of all pairwise distances between the orange (orange line) or blue (blue line) points in (**A**) is characterized by a linearly growing curve; note: the gradient of the curves differs by a factor of 4, since it scales with N^2^ (N = number of points). (**C**) Frequency distribution of pairwise distances between simulated signal points (not shown) organized into clusters in the absence of signals randomly distributed outside these clusters. For this type of distribution, a peak reflecting cluster parameter appears instead of a linearly growing curve. Compared are the curves simulated for clusters of different parameters and different cluster numbers (see the inserted legend or below) within an area of 24,000 × 24,000 nm^2^. R = radius of clusters; N_Cl_ = numbers of clusters; N_P_ = number of points per cluster; a = quadratic area analyzed. Note: the number of clusters is proportional to the area below the curves. (**D**) Frequency distribution of pairwise distances simulated for a strictly clustered point pattern and a clustered point pattern combined with a random signal distribution, respectively. The blue curve reflects the same cluster formation as the blue curve in (**C**) but within an area of 24,000 × 24,000 nm^2^; the orange curve then describes the clustered pattern within an area of 2400 × 2400 nm^2^ (orange) embedded into a random point distribution; note: the smaller area of 2400 × 2400 nm^2^ includes only signal points within a simulated cluster while the larger area of 24,000 × 24,000 nm^2^ also includes signal points in the cluster surroundings. (**E**) Example of a signal point pattern with coordinates of cluster barycenters (orange) visualized together with randomly distributed points (blue); (**F**) Frequency distribution of pairwise distances between points compared for different signal spatial patterns: A homogeneous (random) point distribution (blue) and a distribution for circular clusters with homogeneously distributed points (orange) were created. The orange curve indicates the clustering of orange points around the barycenters of clusters shown in (**E**). The blue line represents the randomly distributed blue points (match with (**A**)). For comparison, both distributions were evaluated simultaneously, i.e., without discriminating the point color (green). The result is different from the outcome where the blue and orange point datasets were evaluated separately and the obtained distribution curves added (red). This difference can be abrogated by adding an additional background (purple dotted). If one adds this background curve to the blue and the orange curve, the result gives the overall distribution seen in real samples (green, pink dotted), which precisely corresponds to the situation where the clustered (orange) and random (blue) signal distributions were evaluated simultaneously (green curve). Note: This figure explains Figure 1E in detail.

Figure 3 visualizes typical results obtained for data generated by Monte Carlo simulations. A random homogeneous distribution of signal points (Figure 3A) leads to a linear increase in the distance frequency histogram (Figure 3B), because the volume of concentric circular shells demarked around any signal point, and therefore also the number of signals at the circular shell, grows with the shell diameter, i.e., the distance from the signal point (Figure 3B, insert). The slope of the straight line is scaled with the number of points (compare the blue and orange curve for datasets of 2000 and 4000 signal points, respectively; Figure 3B). If the points appear in clusters; as, for instance, in the case of IRIF, heterochromatin domain or PML body labeling; smaller distances occur more often. This creates a peak at smaller distances (Figure 3C). The width of the peak gives the maximum distance between two points in the cluster, i.e., the cluster diameter (compare the Russet and orange curve for cluster radii of 50 and 80 nm, respectively; Figure 3C). The area under the peak is a measure of the number of points in a cluster multiplied by the number of clusters (Figure 3C,D). As mutual distances are measured for all possible signal pairs, each distance is counted twice (e.g., for signal points X and Y, both X → Y and Y → X distances are included). Mutual distributions of clusters (as individual entities) can be added during the evaluation. This means that if one looks at the distance analysis of a cluster distribution, a high density of clusters leads to a fluctuating distribution (not shown). The exact shape of the peak then reveals the distribution of points within the cluster, i.e., whether they are homogeneous across the clusters or not. The right-shift of the cluster peak points to a ring-like distribution while the left-shift is characteristic for a more focused distribution. If a background is overlaid (Figure 3E,F), the resulting analysis consists of three parts: (a) a homogeneous distribution (linear); (b) a clustered distribution (peak); and (c) a cross term, which contains the distances between the points of the two components.

### 2.3. Clustering and Persistent Homologies of γH2AX Foci and Heterochromatin

**Figure 4:**
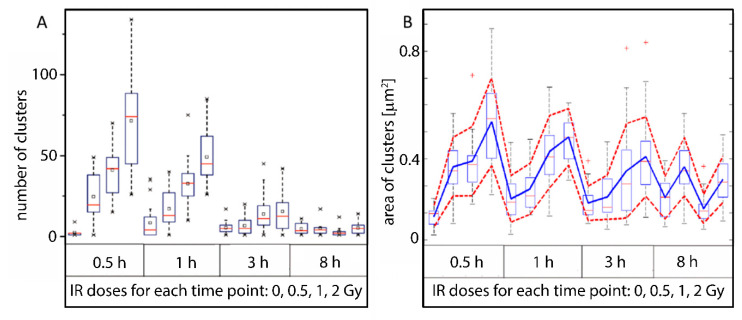
(**A**) Number of γH2AX clusters detected per cell vs. radiation dose and time PI (the figure is reproduced from [58] with permission from the Royal Society of Chemistry). HeLa cells were irradiated with 240 kV X-rays at different doses ranging from 0 (control) to 2 Gy. After irradiation, aliquots of the irradiated cell cultures were fixed at the given time points (0.5 to 8 h PI) and labeled with antibodies against γH2AX. (**B**) Areas of detected clusters calculated for different doses and times PI. The boxplots show the median values (red line), the lower and upper quantile (box), and ± 2 standard deviations from the mean value (dashed bar); the x symbols (panel (**A**)) indicate the maximum and minimum values; the red cross symbols (panel (**B**)) indicate the outliers.

The following data further complement our previous studies [58]. Applying the Ripley distance analysis [82] as described in the simulation above revealed that microscopically visible γH2AX foci consist of three to four (nano)clusters (minimum cluster radius 200 nm; minimum number of cluster points 46). These clusters were obtained after an interactive optimization of cluster parameters. For this process, a spectrum of cluster parameters was applied to the measured data and compared with a random distribution. With the radiation dose, the overall number of these clusters in the cell nucleus increased with the focus number in a way characteristic for classic dose-efficiency curves. During the repair, the number of clusters decreased and reached the level of the non-irradiated control cells at 8 h post-irradiation (PI) (Figure 4A).

Based on the number of points within clusters and their distances (explained in Figure 3), it was possible to calculate the cluster areas (Figure 4B). Two size ranges were found. The γH2AX clusters of the non-irradiated controls were always smaller (approximately 0.09 to 0.15 µm^2^) than those of the irradiated specimens (approximately 0.20 to 0.42 µm^2^), except of the sample analyzed 8 h PI, where more factors are expected to influence the average IFIF size (see the note below) and random fluctuation may appear. Only the smaller sized clusters remained at later repair times (>3 to 8 h) (Figure 4B) when, for the radiation doses and radiation types studied, the average focus numbers are again at control levels. Note: The area value for 0.5 Gy at 8h PI (B) is unexpectedly high while the value for 1 Gy is unexpectedly low compared to the trend observed for all other (shorter) periods of time PI. This could be the result of the interference of DSB repair with other processes that have become already relevant at later PI times, such as replication under conditions of incompletely repaired genomes or even replication stress. In combination with a relatively low number of nuclei studied by SMLM due to extreme technological demands, significant fluctuations of results could occasionally lead to a cell selection bias. One of the possible explanations of the observed paradox is that while the average size of γH2AX clusters increases with radiation dose and decreases with PI time, this decrease is stopped at 8 h PI because irreparable (often clustered and therefore larger) γH2AX clusters preferentially persist in cell nuclei at this period of time. This effect may be more pronounced for lower doses (0.5 Gy in this case) as most DSBs are already repaired “except for” the complex ones. For higher doses (2 Gy), more non-complex DSB lesions may still be present, thus decreasing the average area of foci compared to lower doses. In addition, replication may proceed at 8 h PI after low radiation doses as the cell cycle checkpoint may become unblocked due to only low DSB numbers. As replication-associated γH2AX clusters are smaller compared to clusters induced by radiation and are often far more numerous, the biased selection of nuclei with the replication-associated IRIF foci may lead to a significant decrease of the average size as observed for 1 Gy.

It is well-known that the architecture of chromatin is not random [83,84,85,86]. Importantly, structurally distinct chromatin domains with specific functions show different sensitivity to radiation damage [11,21,52,53,87,88]. Creating additional level of complexity with the potential influence on DSB damage and repair, specific chromatin domains such as euchromatin and heterochromatin are non-randomly distributed in the cell nucleus [84,89,90]. In turn, this means that the nuclear distribution and potentially 3D spatial organization (topology) of γH2AX foci (or IRIFs in general) is also nonrandom, which could have an impact on the accessibility and/or binding efficiency of repair proteins to a given damaged site. Hence, local chromatin architecture at damaged sites and repair focus topology could be expected to participate in the cell decision-making for a particular repair pathway at individual DSBs [21,91]. Indeed, chromatin architecture has been shown to affect regional mutation frequencies [92], which might be caused by the activation of different repair pathways (both due to their different mechanisms and kinetics).

**Figure 5:**
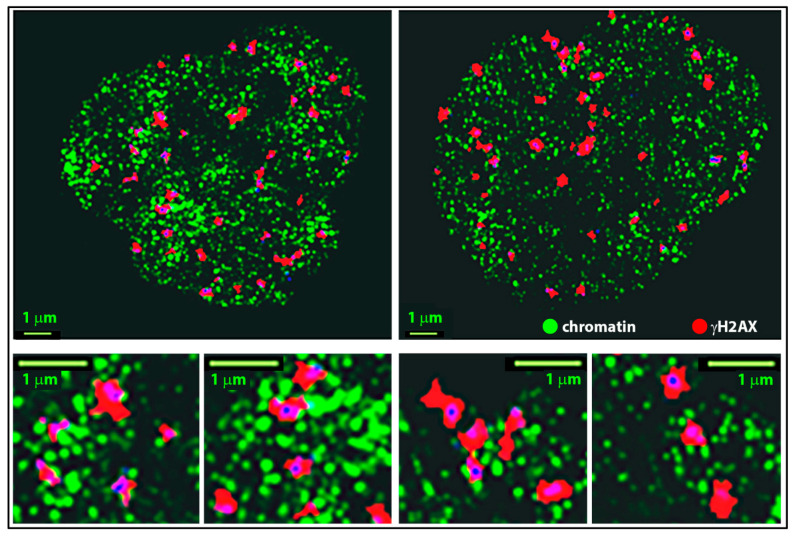
Computed images of two HeLa cell nuclei 30 min after irradiation with 2 Gy X-rays and SMLM of antibody-labeled heterochromatin (green) and γH2AX (red). The artificial images were prepared from the localization matrices (i.e., matrices of signal coordinates and localization errors) obtained for green and red signals. The intensity of the green signals is encoded by the next neighbor density of signals (clustered signals, herein the chromatin domains, are therefore brighter) whereas the red signals are merged into coherent clusters. Blue signals correspond to signal overlap of the two channels. Note that these images were artificially prepared from point data of two color channels by mathematical data processing (see Figure 1 for general description of the whole procedure). Note: Parts of this figure are shown in Figure 1I.

**Figure 6:**
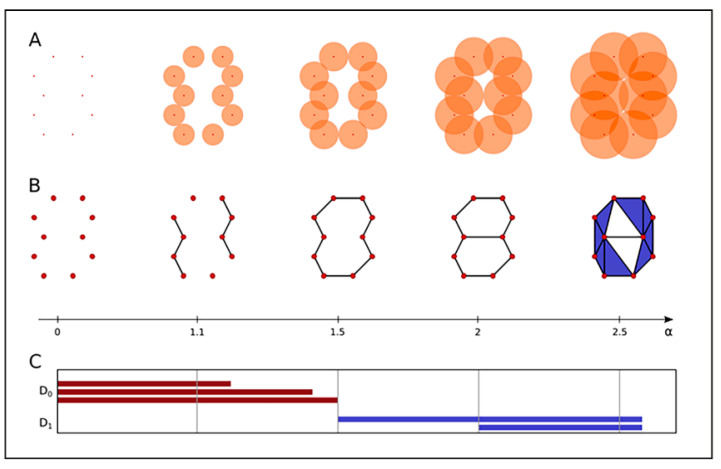
Persistent topology analysis and the barcode data representation. (**A**) Continuously growing spheres (orange) around the signal point data, exemplarily shown for five different radius (α) scales, illustrate the idea of the α-shape filtration (i.e., “component” formation). (**B**) As the growing spheres mutually contact each other, the corresponding sphere centres (i.e., the involved signal points) are connected by an edge. Whenever a triangle is formed, it is included in the complex as a face element. (**C**) Barcodes (Betti numbers) of dimension 0 (D_0_) and 1 (D_1_) corresponding to connected components (dark red bars) and holes (blue bars). For detailed explanations, the article by Hofmann et al. [79] is recommended. Note: This figure explains Figure 1F in detail.

Recently, we have introduced a novel approach to analyze γH2AX (IRIF) clusters by their topology to determine similarities of these clusters depending on their association with heterochromatin or non-heterochromatin [61,79]. Based on the pointillist localization data obtained by SMLM for heterochromatin (labeled by anti-H3K9me3 antibodies) and γH2AX clusters (Figure 5), an analytical method for cluster characterization by means of persistence homology (Figure 5) was applied [61,79,93]. Using established calculations and mathematical operations, the method allows to compare the distributions of two point datasets (here the point distributions of γH2AX clusters associated with heterochromatin and non-heterochromatin, respectively) in a cell-independent manner; the calculations thus provide a mathematical measure of the γH2AX cluster similarity in the dependence of a specific chromatin environment (Figure 5 and Figure 6).

For topological calculations, a minimum cluster radius of 200 nm and a minimum point number of 50 was used to define γH2AX clusters. The centers of the γH2AX clusters were computed with the surveyors area formula [94]. These centers were used as the barycenters of circular shells in which the heterochromatin density was calculated (the heterochromatin density equals the number of heterochromatin labeling points per area of a shell). Based on this density distribution, the γH2AX clusters were divided into heterochromatin-associated (HC) and non-heterochromatin-associated ones (nHC) (for details of the calculation, see [61,79]).

A major principle of “topological analysis” is to record properties of point patterns, which are invariant under certain deformations of the object. This is a fundamental advantage as nuclei and also individual objects within nuclei, such as IRIFs, are differently oriented in space and differently deformed. Mathematically, these deformations correspond to continuous transformations of the topological space defined by the structures. In the context of the application described here, the attention will be focused on two properties: (a) the number of “components”, which are independent from each other in such a sense that connections between signal points only exist within the respective components; and (b) the number of “holes” of the structures within the components (Figure 6). In algebraic topology, these properties are called the Betti numbers for zero and one-dimensional simplicial complexes [93]. They are the topological invariants that distinguish between different topological spaces.

SMLM images are signal point-sets for which components and holes are defined (Figure 6A,B). A geometric relationship among the points within a signal cluster (e.g., one IRIF focus) is defined by growing spheres of radius α around each of clustered points. Whenever two spheres mutually contact each-other, these centers of the growing spheres (i.e., the two involved signal points) are connected by an edge. Connected points then belong to the same component. With increasing radii of the spheres, more and more points are connected to previously disjointed components, leading to their gradual joining. Thus, the number of components decreases with an increase in the radius α. At the end of the procedure, all components are combined into a single component for each γH2AX cluster. For the definition of holes, the simplest polygon, the triangle, is appropriate. Whenever three edges form a triangle, the area of the triangle is considered and the holes are counted.

The results are presented as “barcodes” [95] to track the formation and disappearance of components and holes with increasing α (Figure 6C). These barcodes contain information about components and holes in a compact and illustrative way, offering the easy mathematical comparison of different sets of barcodes and defining the parameters describing their similarity. The procedure used for quantifying the barcodes’ similarity is based on the Jaccard index [96], as was described in detail in [61,79,93]. The result of this similarity measure is a value between 0 and 1, where a value of 0 means no overlap of two bars and 1 means the identity of two bars. The overall similarity of barcodes of different dimensions (components and holes) is defined as the average of these individual similarity values. Importantly, topological comparisons are independent of the scale so that it is possible to compare variably large foci as well as micro-, meso-, and nano-scaled objects.

**Figure 7:**
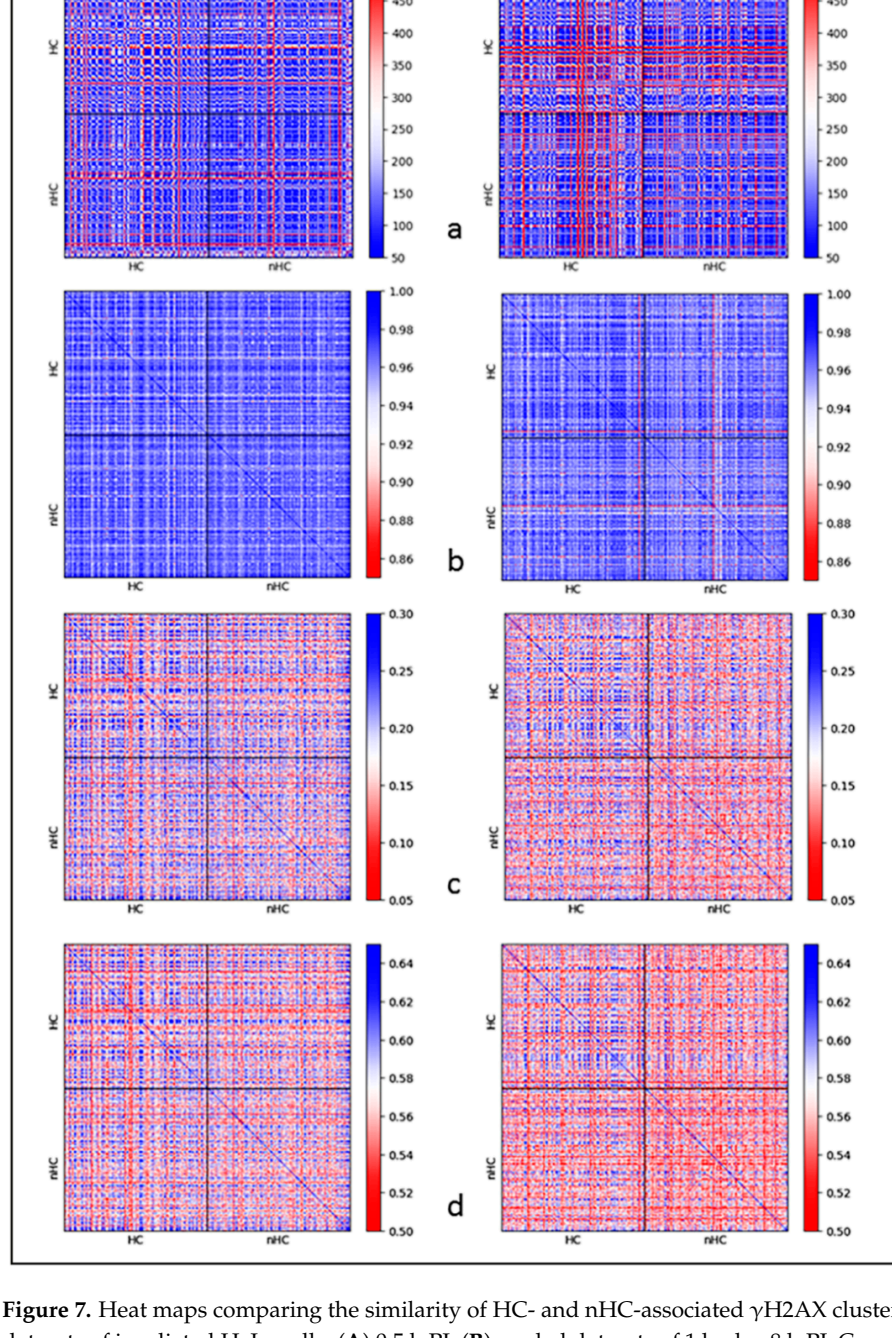
Heat maps comparing the similarity of HC- and nHC-associated γH2AX clusters for two datasets of irradiated HeLa cells. (**A**) 0.5 h PI; (**B**) pooled datasets of 1 h plus 8 h PI. Comparison of sizes (**a**); topologies of dimension 0 (=components) (**b**); topologies of dimension 1 (=holes) (**c**); and averaged topology similarity measures for the dimension 0 and 1 (**d**). In these heat maps, the upper left quarter mutually compares all the HC-associated clusters and the lower right quarter mutually compares all the nHC-associated clusters. The upper right and lower left quarters then both compare nHC-associated clusters with HC-associated clusters. The arrangement of the individual HC- and nHC-associated clusters along the x and y heatmap axes is random but the order of clusters is the same for both axes; hence, a blue diagonal of topological identity can be seen, wherein the individual clusters are compared to themselves. The color codes of the heat maps mean red for dissimilarity and blue for similarity. As a measure of the similarity of the size, the difference in the size of two clusters was used so that a small difference corresponds to great similarity. Data for 0.5 Gy and 1.0 Gy X-rays were pooled into one dataset to increase statistical significance as the results were shown not to depend on dose. It can be seen that γH2AX clusters are mutually more similar in the shorter PI period (0.5 h) and also if they “colocalize” with heterochromatin (compared to euchromatin). See the text for details. Note: This figure is a typical example for the illustration in Figure 1H.

Figure 7 presents the results of topological similarity measurements for HC- and nHC-associated γH2AX clusters formed in HeLa cells at different PI times upon their exposure to 0.5 Gy and 1.0 Gy of X-rays, respectively. Since the results did not show dose dependent differences, data of both doses were merged.

The cells usually start repairing the DNA damage caused by single- (SSBs) and double-stand breaks (DSBs) immediately post-irradiation so that the radiation-induced γH2AX foci or focus clusters, respectively, can be seen in some cases as early as 5 min PI. Hence, to obtain information for all early, mature, and late foci, the cells were fixed at 0.5 h, 1 h, and 8 h PI. Cells in the first dataset contained 1768 clusters and were analyzed at the repair time of 0.5 h PI (Figure 7a). In the second dataset, the repair times of 1 h and 8 h PI were merged in order to compensate for decreasing cluster numbers due to ongoing repair and achieve comparable cluster numbers (herein 1618 clusters) for both datasets (Figure 7b). The similarity in size between these two groups (HC- and nHC-associated) of γH2AX clusters was determined first (Figure 7, panels a). Since the clusters are polygonal, the root mean square of the distances of the points of the convex hull has been defined as a measure of cluster size. The results of the topological similarity measures are then depicted as heat maps in panels b–d (Figure 7(Ba,Bb)).

**Figure 8:**
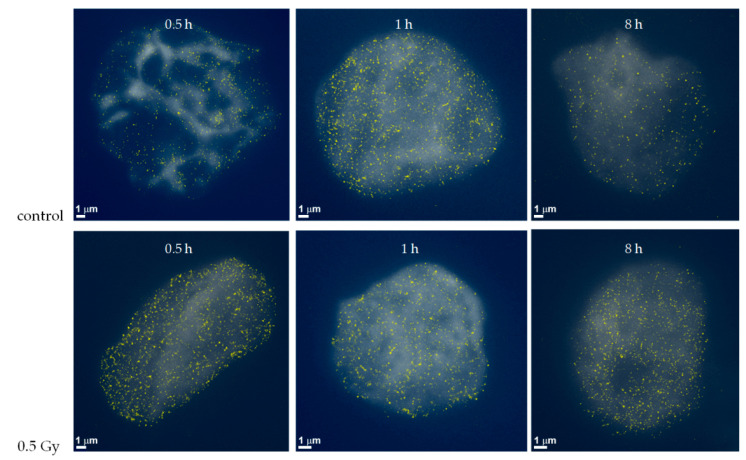
SMLM images of the distribution of H3K9me3 antibody signals in non-irradiated (control) HeLa cells (upper row) and cells exposed to 0.5 Gy X-rays (lower row). Examples of cells fixed at different times post-irradiation (0.5 h, 1 h, and 8 h PI) are shown. The H3K9me3 histones detected by SMLM are visualized as yellow dots and, in the background, the widefield image of the H3K9me3 staining is shown.

The observations presented here confirm our findings for another cancer cell type [61,79]. Studies on different cell types are important as IRIF formation kinetics and structural (e.g., size) and potentially topological parameters seem to depend on the cell type. The sizes of the radiation-induced γH2AX clusters were about the same for the two X-ray doses considered (0.5 Gy, 1.0 Gy) and the association with heterochromatin did not influence this parameter either. The focus size uniformity measured here supports the results obtained by Ripley’s distance analyses (Figure 4b). On the other hand, the proximity of γH2AX clusters to heterochromatin had a significant impact on their (nano)architecture. For dimension 0, all clusters showed about the same similarity of the components of interconnected points (Figure 7, panels b). However, when the barcodes of dimension 1 (representing the holes) were compared, the HC-associated clusters revealed a higher similarity to each other, compared to their nHC counterparts (Figure 7, panels c). Similar results leading to the same conclusion were obtained also for the average similarity of components and holes (Figure 7, panels d). The reason for this observation has to be studied. We can hypothesize that genetically inactive heterochromatin shows a rather homogeneous topology/architecture throughout the nucleus whereas the topology/architecture of active chromatin is more variable due to high differences in the genetic activity of each particular locus. These results also suggest that topological and architectural features of damaged chromatin domains may influence the formation of IRIFs, and thereby contribute to the activation of a particular repair pathway at each individual DSB site. In accordance with this statement, HC- and nHC-associated γH2AX clusters mutually differed in their topology more prominently at 0.5 h post-irradiation than in the later PI times. However, the spatial organization of additional repair proteins in different cell types should be studied to confirm this hypothesis. In order to explore the topological conditions of the surrounding heterochromatin, the coordinates of the detected fluorophores attached to H3K9me3 antibodies were determined. These data define a signal point cloud for each nucleus (Figure 8). By means of persistent homology evaluation, a barcode representation was computed (Figure 6) over which geometrical scales the topological objects, i.e., components and holes, persist for each point cloud. Due to the high number of points considered in the cloud of each nucleus, the comparison by Jaccard indices and heat maps would exceed the current computing capacity. Thus, a different approach was applied for this comparison: The barcodes (i.e., the α shape filtration and the persistence of the components and holes; Figure 6) were obtained using the python package “gudhi” [97]. The values for the birth (beginning of a bar) and death (end of a bar) of components and holes were rounded to two decimal places and the entries for which the birth values coincided with the death values after the rounding procedure were excluded (in order to compute the mathematical operation for the data acquired).

**Figure 9:**
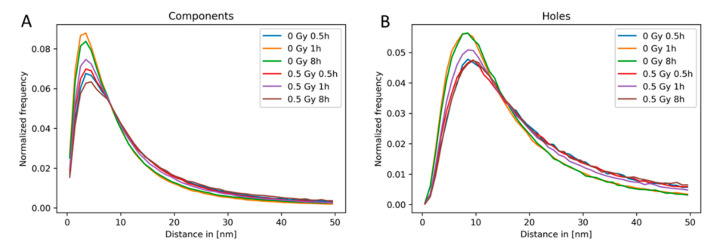
Relative frequency of the endpoints of dimension zero and dimension one bars (for details, see Figure 6 and the explanation in the text). The normalized histogram of the distribution of the endpoints of the bars representing the persistence of (**A**) components and (**B**) holes is shown for non-irradiated HeLa cells and samples irradiated with 0.5 Gy X-rays and fixed at different time points post-irradiation. For each treatment, the average of the normalized histograms of the different nuclei is shown.

The “separation of the sub-components” [98] and the size of the holes can be obtained by considering the endpoints of the bars of components and holes. Hence, for each point cloud defined by the H3K9me3 blinking events detected in a nucleus, a normalized histogram showing the distribution of endpoints of the bars (=the highest α value of a bar) of components and holes was computed. The average distribution of the endpoints of bars within all nuclei of the given sample is shown in Figure 9 for non-irradiated cells in comparison to irradiated cells analyzed at different times PI.

At relatively early post-irradiation times (30 min PI), neither the distance distributions of the endpoints of the components nor the distance distributions of the endpoints of the holes differed for the irradiated and control cells. At later PI times, the distributions of the endpoints of the components and holes were nearly equal for control cells at 1 h and 8 h PI. However, the distributions for the irradiated samples differed considerably from the non-irradiated ones at both these time points. The holes appeared to be more open after irradiation as compared to the controls (Figure 9A,B). These results indicate a post-irradiation reorganization of the heterochromatin.

### 2.4. Clustering of Nucleosomes upon Cell Exposure to Different Radiation Quality

We applied Ripley’s clustering analysis to explore radiation-induced chromatin reorganization during DSB repair by investigating the organization of intrinsic histone staining in the HeLa cells, which were stably transfected for expressing green fluorescent protein (GFP)-labeled histone H2B (H2B-GFP) [44,52,99]. For irradiation, a mega-voltage generating linear accelerator (LINAC) (Elekta™ Synergy system with Agility^®^ beam shaping and a flattening filter) and a kilo-voltage generating INTRABEAM™ IORT machine were used. Both machines are typical radiation sources in clinical cancer treatment. From the Linac, the 6 MV photon beam was chosen to irradiate the cells, while the energy of photons from the INTRABEAM™ machine was fixed at 50 kV. The INTRABEAM™ system delivered the radiation at a constant dose rate of 258 mGy/min; the Linac was run with a dose rate of 300 mGy/min. A dose of 3.5 Gy was delivered with both systems.

**Figure 10:**
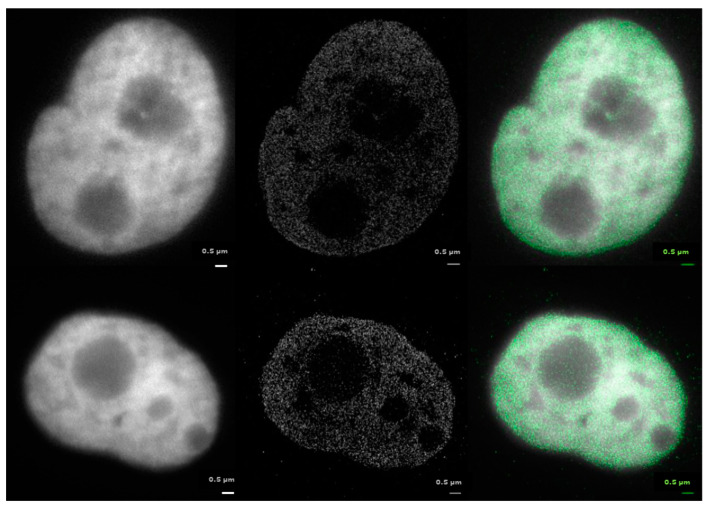
Examples of HeLa cell nuclei with H2B histone of nucleosomes labeled by green fluorescent protein (GFP). Left: widefield microscopy images showing the typical, diffraction-induced spreading of the signals; middle: the same nuclei visualized based on the localization (SMLM) data; right: overlay of the left and the middle images.

The aim of this study was to analyze changes in the nuclear nanostructure caused by early and late repair mechanism processes after irradiation. For this purpose, the HeLa cells were fixed with formaldehyde at four different times after irradiation: 15 min, 30 min, 12 h, and 48 h PI. About 15 cells were recorded for each repair time. About 20,000 signals of H2B-GFP were observed on average in the cells by SMLM (see examples in Figure 10, middle images). A threshold range from 10,000 up to 50,000 signals detected per cell was set as a selection criterion before statistically analyzing the cells by the clustering algorithm.

In order to quantify the chromatin rearrangements during the repair after irradiation with the same dose but considerably different energies and dose rates, clusters with more than three fluorophores (points) located next to each other in a circle with the radius of 40 nm were determined. Based on the point densities and density fluctuations, the maximum number of points in a cluster was set to 100 and the maximum distance between the points within the cluster was fixed at 200 nm. In parallel to irradiated cells, the clustering information was obtained also for a control set of non-irradiated HeLa cells labeled and treated in the same way as irradiated cells, and fixed after 48 h of culturing. This fixation time refers to the longest period of repair time analyzed in irradiated cells and was used to exclude that cell culturing may have influenced the results (e.g., via chromatin re-arrangements potentially associated with cell cycle progression/cell proliferation). For this non-irradiated cell population, 13% of the signals in each cell fulfilled the cluster criterion. The information obtained from the clustered fluorophores was also compared to a set of random data generated by the algorithm using the localization information (number of points detected etc.) provided in the data set.

**Figure 11:**
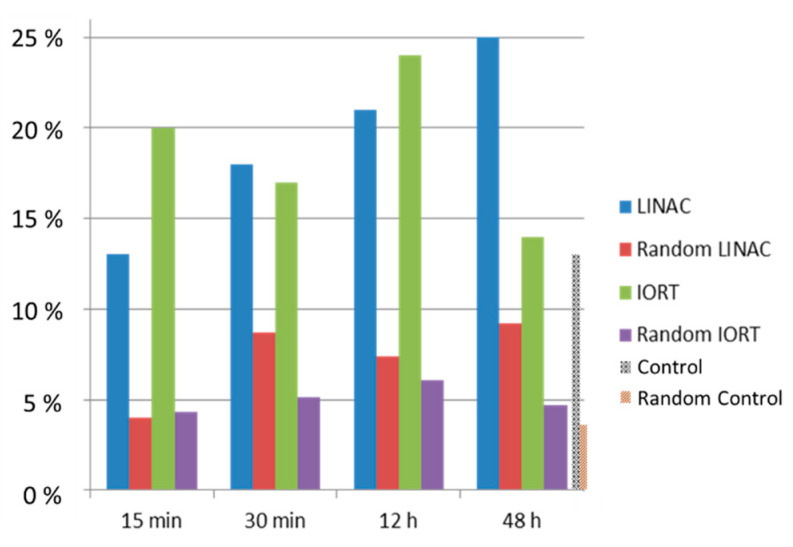
Nucleosome cluster formation after irradiation of GFP-H2B-labeled HeLa cell nuclei with a dose of 3.5 Gy (X-rays) at energies of 6 MeV (LINAC) or 50 keV (IORT). Comparison to random H2B distribution is also provided. In the non-irradiated control cells (shown at 48 h of cultivation), 13% of signals followed the cluster criterion. See text for further details.

The results of the cluster analysis (Figure 11) revealed an increase in the amount of clusters during repair, which significantly varied from randomly arranged points. Importantly, the responses differed for the two given qualities of radiation. After the low-energy irradiation, the formation of clusters was more variable than after the high-energy irradiation. Additionally, while the number of clusters in cells exposed to low-energy radiation dropped to the value measured for the non-irradiated control after 48 h, a continuous rise was observed for the high-energy irradiation during the whole post-irradiation period of time investigated.

## 3. Discussion

After reaching the physical limits of confocal microscopy, a detailed analysis of IRIFs and surrounding chromatin using super-resolution methods becomes essential for the study of the mechanisms of chromatin damage, repair, and rearrangement (mis-repair) [58,61,81]. Among super-resolution techniques, single molecule localization microscopy (SMLM) used in the present manuscript offers several advantages, in particular the ability to evaluate results mathematically and study the same samples in parallel by confocal microscopy [10,11,58,61,67,79]. As already mentioned, SMLM allows the direct measurement of the mutual spatial relationships between signals of individual investigated molecules and the determination of the number of these molecules; hence, we can apply SMLM to visualize differently defined protein clusters and their properties and quantify their topology and molecular composition.

SMLM data result in a signal point cloud that can be analyzed according to mathematic approaches, known in geometry and topology. Thus, an abstract reduction of the point cloud to distance frequency histograms or barcodes representing persistent homologies of different dimensions is a robust novel approach to quantify and compare biological structures/objects without the need for image analysis tools. In the present manuscript, geometric and topological similarities were elucidated for γH2AX foci and chromatin, paving the way for gaining new insights into presumptive physical mechanisms controlling the access of repair proteins to DNA damage sites and thus repair pathways. The results currently available suggest that there is a relationship between the architecture of IRIFs and the architecture of chromatin at DSB damage sites. This relationship points to a non-random character of IRIF assembly and, in turn, a (functional) role of chromatin (genome) architecture in cell response to DNA damage by environmental or therapeutic factors [21]. In addition, we have recently described differences in the IRIF nanoarchitecture for normal human skin fibroblasts and highly radioresistant U87 glioblastoma cells [55]. In this work, we show that the alteration of IRIF nanoarchitecture could be a more general phenomenon in cancer cell biology. While general principles of chromatin organization remain preserved in cancer cells, cancer cells (with HeLa cells studied in the present work being an excellent example) are characterized by local genome reorganizations, amplifications, and epigenetic changes leading to local alterations of gene expression and changes of higher-order chromatin architecture [100]. Hence, this increased variability of chromatin structure could be reflected by a higher variability of IRIF nanoarchitecture in cancer cells. Presupposing a functional relevance of IRIF topology, it is possible that IRIF nanoarchitecture adds an additional layer of complexity to the deregulation of DSB repair in cancer cells and may explain differences in the repair capacity or repair mechanism preferences of different cancer cell types without obvious changes in repair protein expression or functionality. Downregulation, upregulation, or mutations of repair proteins in cancer cells could further augment the variability and deviations of the IRIF nanoarchitecture from the functional nanoarchitecture in normal cells. Altered IRIF nanoarchitecture may then reduce the reliability of canonical repair pathways or prefer alternative error-prone repair mechanisms, thus supporting the generation of errors during DNA repair, as is often seen in cancer cells.

The presented findings, together with the above-mentioned fact that chromatin architecture is altered in tumor cells [92], also allows us to assume that super-resolution (SMLM) studies may reveal individual differences in the nanoarchitecture of IRIFs and/or chromatin [92], which could be predictive of individual radiosensitivity/DNA repair capacity and used for personalized diagnostics and therapy of cancer [92]. However, nanoscale investigations of chromatin architecture reorganization after radiation damage and during DSB repair are at the very beginning. Clinical applications will require specialized hardware and software that is not yet available for routine cancer research and diagnostics.

Nevertheless, the above-described approach is unusual for microscopy and microscopy users being familiar with “standard” image interpretation may doubt the results and their meaning. Simulations, as shown in Section 2.2, with well-defined input data may be helpful to overcome such doubts. In addition, mathematics has proven the results analytically so that the transfer to biophysics is no doubt possible and reasonable.

The nano-scaled results brought about by SMLM depicted microscopically detectable foci as associations of nanoscopic (sub)foci (or nano-foci) of γH2AX molecules. The mutually similar organization of γH2AX sub-foci gives a hint that the topology of microscopic γH2AX foci is not random [58,61,79]. Whether this internal nanostructure of γH2AX foci reflects a defined functional arrangement compatible with one or another repair pathway or even participates in the decision-making process for a particular repair pathway at each individual DSB site has to be further studied. The observation that γH2AX foci located in heterochromatin are mutually more similar than γH2AX foci located in euchromatin, described in the current manuscript and our previous work [58,101], is well compatible with this hypothesis.

Moreover, in addition to offering new insights into the nanoarchitecture of IRIF and focus-surrounding chromatin, the present observations illustrate the urgent need for a multi-scaled analysis of biological objects [21,67,102,103]; this means that future (high-resolution) confocal microscopy approaches might be mutually correlated with SMLM results. The nano-scaled data can help to mechanistically understand cellular processes observed at the microscale, and, in turn, unprecedented and sometimes difficult to grasp SMLM data can much more easily be interpreted if it is possible to place them in the context of already known microscopic objects and events. It should be emphasized that the nanoscale structures and processes are not only the microscale structures and processes “understood” in much better detail. At the nanoscale, various processes may proceed without being distinguished at the microscale due to their mutual interference leading to a seemingly chaotic microscale image. On the other hand, this interplay between nanoscale processes may give rise to more complex entities or processes that are unique for the “micro-world”. Hence, we consider the strategy based on the correlated confocal microscopy, SMLM microscopy, and the mathematical evaluation of data based on the persistent homology (that is, invariant to different scales) to be one of the best ways to approach the correct conclusions when studying multi-scale processes such as DNA repair. Hence, the concomitant use of confocal microscopy and SMLM on the same samples can help to understand the newly emerging nano-world.

## 4. Materials and Methods

### 4.1. Cell Culturing and Preparation for Irradiation

For the experiments, a HeLa cell line was used with H2B-tagged green fluorescent proteins (GFP), originally developed by Tobias A. Knoch [99]. The genes for tagging histones were inserted into the multiple cloning sites between the Hind-IIIc region and the start codon of the AFP gene, using plasmids based on the pECFP-1 plasmid. The transfection of cells was carried out using the Lipofectamin transfection kit from Gibco^®^ (ThermoFisher Scientific, Waltham, MA, USA).

The H2B-GFP-expressing HeLa cells [44,58,99] were cultured in RPMI 1640 medium containing 10% fetal bovine serum. The adherent cells were incubated at 37 °C with 5% CO_2_. The cells were grown for one week (with subsequent passaging) before being ready for the irradiation experiments. About 15,000 cells were seeded on sterile glass slides and cultivated for three days for YXLON Maxishot irradiation. At given time points post irradiation, the cells were washed in Hank’s balanced salt solution (HBSS) (Gibco) and fixed in 4% acid-free formamide (C. Roth, Karlsruhe, Germany) in HBSS, each for 5 min at RT. Preparations were then washed in 0.5% glycine in PBS for 3 min and extracted for 20 min in 0.5% TritonX100/PBS (C. Roth). Immunostaining with mouse anti γ-H2AX antibodies (Merck Chemicals, Darmstadt, Germany) marking DSB damage sites, and rabbit anti-H3K9me3 (Abcam, Berlin, Germany) marking heterochromatin was carried out as described [60,94]

To prepare the cells for irradiation via the Linac or INTRABEAM™, the cells were cultured on circular coverslips of 10 mm diameter and with a thickness of 1.5 mm. The coverslips were placed in sterile 35 × 10 mm^2^ petridishes with four internal wells where four separate coverslips could be placed. The growth area available for the cells on each cover slip was about 93 mm^2^. To prevent the clumping and stacking of cells, a minimum of 15,000 cells and a maximum of 20,000 cells were allowed to grow on the cover slips. To make it easier to image the cells, they were grown till the cover slip was about 70 to 80% confluent.

To transfer the live cells to the irradiation machines located in the hospital, the petridishes were closed with Parafilm^®^-M and placed in a Styrofoam box containing ice. The ice temporarily slows down most of the cellular processes, thereby allowing most of the cells to remain in the same cycle of growth and division that they were in while inside the incubator. In the case of longer irradiations, the pH was maintained because of the HEPES, which was added while preparing the medium.

Immediately after irradiation, the petridishes were kept on ice for 10 min, which was utilized to transfer the cells from the machines to the cell culture laboratory. After 10 min, the Parafilm™ was removed and the petridishes were incubated at 37 °C with 5% CO_2_. For microscopy, the cells were fixed in 4% formaldehyde freshly prepared from paraformaldehyde. The fixed cells on the coverslips were embedded in ProLong^®^ Gold anti-fade reagent.

### 4.2. Irradiation

(a) For foci and heterochromatin analyses, the cells were irradiated at room temperature with 240 kV X-rays filtered with 3 mm beryllium at a dose rate of 1 Gy/min at 13 mA (YXLON Maxishot, Hamburg, Germany). The absorbed dose was measured with a PTW Unidos dosimeter (PTW Freiburg GmbH, Freiburg, Germany). The cells were irradiated with doses of 0.5 Gy, 1 Gy, and 2 Gy. Control cells were sham-irradiated.

(b) For whole chromatin analyses, the samples were irradiated by a mega-voltage generating linear accelerator (Elekta™ Synergy system with Agility^®^ beam shaping and a flattening filter) and a kilo-voltage-generating INTRABEAM™ IORT machine, which were available at the Department of Radiation Oncology, Universitätsmedizin Mannheim. From the Linac, the 6 MV photon beam was chosen for irradiating the cells, while the energy of the photon from the INTRABEAM™ machine was fixed at 50 kV.

The INTRABEAM™ system delivers the radiation at a constant dose rate of ~250 mGy/min. To maintain uniformity in the dose rate delivered to the HeLa cells in both cases of irradiation, the Linac was run on service mode and the dose rate was minimized to the lowest stable setting possible, which was around 30 M.U./minute (300 mGy/min).

### 4.3. Single Molecule Localization Microscopy (SMLM)

SMLM was performed using an in-house built localization microscope [58,67]. Enhanced thermo-mechanical stability and high-precision optical elements led to a localization precision for a given fluorescent point of about ±10 nm while the temperature drift was below 1/100 °C at 23 °C room temperature. For illumination, the light path was equipped with a LightHub laser combiner (Omicron Laserprodukte GmbH, Rodgau-Dudenhofen, Germany) for four laser lines (405 nm, 491 nm, 561 nm, 642 nm) and the wavelengths were separated by a polychromatic AOTF (AA Opto Electronic, Orsay Cedex, France). The laser beams were shaped by a variable beam expander 10BE03-2-8 (Standa ltd, Vilnius, Lithuania) and a Flat-Top-Profile forming optics—PiShaper (AdlOptica GmbH, Berlin, Germany). The circular Flat-Top laser beam profile was focused into the object plane using an achromatic focusing lens (f = 250 mm) and a 100×/NA 1.46 oil plan apochromatic objective lens (Carl Zeiss Microscopy, Göttingen, Germany). The fluorescence light was separated from the illumination light by two quadband interference filter glasses F73-410 and F72-866 (AHF Analysentechnik AG, Tübingen, Germany). The emitted light was magnified by the objective–tube lens pair (Carl Zeiss Microscopy, Göttingen, Germany) and a twofold expander before being detected on an Andor Ultra EMCCD (iXonUltra 897, Andor Technology, Belfast, Northern Ireland) with a pixel size of 16 × 16 μm^2^. For each dose and repair time point, 40 cells were recorded and evaluated by home developed software (see above).

## 5. Conclusions

Microscopic analyses of γH2AX nuclear foci/clusters formed at DSB sites with the participation of a number of other repair proteins, as, for instance 53BP1, MRE11, pATM, Rad51, BRCA1, etc. (e.g, [53,104,105]), have contributed significantly to the current understanding of radiation-induced DNA damage, repair and misrepair processes (see, e.g., our earlier works [11,21,52,106,107,108,109]). In addition to the biochemical processes taking place at DSB sites, microscopy discovered important relationships between the damage-surrounding chromatin architecture, DSB generation and repair mechanisms, and mechanisms of chromosomal aberration formation following the exposure of cells to various types of ionizing radiation [110]. Studying the organization of DNA repair processes in space and time on the micro-scale would not be possible without microscopy. Circumventing the Abbe diffraction limit and developing various methods of super-resolution microscopy then push the architectural and structural analysis of IRIF and repair processes to the molecular level [21,58,67], which is a milestone in cell biology and radiobiology. It will undoubtedly be extremely exciting to see how nanoscopic studies broaden and potentially change our understanding of the mechanisms of NHEJ, HR, alternative repair pathways, and the mutual regulation of this complex repair network.

## Data Availability

Supporting results can be found in [58] or on request to the corresponding authors.
